# Clinical Lectures and Cases in the Wards of Illinois General Hospital

**Published:** 1851-03

**Authors:** N. S. Davis

**Affiliations:** Prof. Principles and Practice of Medicine in Rush Medical College, and one of the Physicians to the Hospital


### ARTICLE VI.

*Clinical Lectures and Cases in the Medical Wards of the Illinois General Hospital.* By N. S. DAVIS, M. D., Prof. Principles and Practice of Medicine in Rush Medical College, and one of the Physicians to the Hospital. Reported by B. F. WHITE, Interne.

CASE 1st. T. Y., Male, aged about 35 years, admitted this day Dec. 9th, 1850; occupation a sailor on the Lakes. The patient before us, remarked the doctor, is a strong laboring man, in the middle period of life, and externally presents us with a flushed face of arterial hue, an active or excited expression of countenance; a hot and dry skin; a hurried, laborious, and painful respiration; the pain sharp and severe, and restricted chiefly to the left axillary and sub-axillary regions; cough frequent, painful, and suppressed; expectoration pretty free, and consisting of a red, liquid mucous which is generally known as the bloody or *brick dust sputa.* The patient complains of great oppression or sense of fullness and tightness across the chest, and of a heavy sore pain in the frontal region, much aggravated by coughing. The pulse is 90 per minute, full, and only moderately firm; there is much thirst; the tongue covered with a whitish fur; and the bowels slightly costive.

The position of the patient is dorsal, with the head and shoulders somewhat elevated. These symptoms, continues the Dr., are fairly and fully characteristic of inflammatory or symptomatic fever, dependent on active inflammation in some important organ; and the acute pain located in the lateral part of the chest, the difficult breathing, and the red or bloody expectoration sufficiently point to the Lungs as the suffering organs. But they neither inform us of the precise location, extent or stage of the disease, nor of the particular tissues involved. It is true, that the color of the expectoration is regarded as diagnostic of pneumonia or inflammation of the air cells and parenchyma of the lungs; while the acute lateral pain, and suppression of respiration and cough are much more characteristic of Pleurisy. For more precise information, howevever, we must resort to the means of physical diagnosis. As I place my hands on the two sides of the chest, you see that it is broad and full, but the right side moves much more freely than the left during each inspiration. There is no enlargement or contraction of either side appreciable to the eye alone. By preserving silence, you will distinguish a pretty fair degree of resonance on percussion over the whole of the right lung, the upper lobe of the left, and the greater part of the middle lobe also, especially anteriorly. As we pass the inferior border of the pectoralis major towards the axilary region we get decided dulness, increasing as we go downwards and backward until we reach the attachment of the diaphragm to the walls of the chest. Indeed, as we cause the patient to turn on the right side and extend our percussion posteriorly, you perceive the dulness occupying both the axillary and subaxilary regions, and the space below the margin of the scapula on the left side.

This degree of dulness occupying the lateral and inferior part of the chest, accompanied by the active febrile symptoms, already detailed, indicates one of two pathological conditions, viz: the accumulation of a fluid in the cavity of the pleura, separating the lung from the thoracic parieties, or inflammatory infiltration into the pulmonary texture, constituting hepatization. Or we may have both these conditions existing in the same case, the consequence of a a pleuro-pneumonia.

On applying my ear now to different parts of the chest, I find the respiratory murmur in the right side and the upper part of the left, simply exaggerated or *puerile*, as it is termed; over the middle lobe of the left side there is a variable degree of coarse mucous rhoneus, and on a level with the lower edge of the Pectoralis major muscle, a pretty distinct vesicular or crepitant *rattle*, while as we pass into the axilla, we loose all sound except a moderate whiffing or tubular murmur produced by the air passing in the larger branches of the bronchial tube. While the patient is coughing or articulating sounds, as by counting aloud, with my ear over the left axillary region, I hear an obscure and somewhat vibratory transmission of the voice, rather intermediate between ordinary broncophony and œgophony. You will have noticed that the dulness on percussion is most complete in the most dependent part of the left side.

Now if we compare these signs with the ordinary symptoms previously stated, we shall be able to draw some very clear and reliable conclusions. Thus, the more or less complete dulness on percussion over the axillary and sub-scapular regions, with distinct *vesicular* murmur somewhat intermixed with coarse mucous rhoncus along the upper margin of the dulness, taken in connection with the laborious and painful breathing, the bloody expectoration, and the active febrile symptoms, leave no doubt of the existence of pneumonic inflammation occupying the greater part of the lower lobe of the left lung, which has passed already into the second stage, or that of *hepatization* in the part first attacked, while it is still actively extending as indicated by the vesicular murmur on the margin or circumference of the diseased part. But this is not all. The acute lancinating nature of the pain, located in the extreme lateral part of the chest, coupled with the most complete dulness in the lower or most dependent part of the side affected, and the entire absence of all respiratory sounds at the same point, render it very certain that the inflammation implicated early, the pleural covering of the diseased lobe of the lung, which has resulted in a moderate amount of pleuritic effusion.

Our diagnosis now becomes plain and definite. The patient is laboring under an acute *pleuro-pneumonia*, implicating chiefly the lower lobe of the left lung.

In regard to the Prognosis, you must consider, first, the length of time since the onset of the disease. The patient says he was attacked four days since with chilliness, followed by high fever, cough, pain in the chest, and all the symptoms before stated; and that they have continued unabated until the present time. You are aware that the length of time required for pneumonic inflammation to run through its successive stages, of congestion, infiltration or hepatization, and suppuration or resolution, is very various, according to the age and previous condition of the patient's health, and the activity of the disease. In childhood, the first stage is much more protracted, than in the middle and later periods of life. In the case before us, the four days it has continued, have given ample time for the parts first affected to have passed into the second stage of the disease. While the parts immediately surrounding those, or at least above, are still in the first stage, as indicated by little or no perceptible dulness and plain vesicular murmur or crepitation. Second—the stage of the disease. As a general rule the longer pneumonia or pleurisy has continued without adequate treatment, the less favorable will be the prognosis. Third: You must note the location and extent of the disease. Inflammation, fully formed, affecting the greater part of one lung, must always be regarded as dangerous. If both lungs are affected the danger is greatly increased.

Again, inflammation of the same extent and intensity, affecting the upper and middle lobes, is more serious than if located in the inferior lobes. In this respect the case before us is favorable. And if we add to this the previously good health, the vigorous sanguine temperament, and the present condition of the pulse, and muscular strength of our patient, we shall predict, that with judicious treatment he will recover.

But in what shall this judicious treatment consist? Before answering this question you should see clearly the indications to be fulfilled. Now what are the objects to be accomplished in the case before us? I answer, first, to arrest the determination of blood to the inflamed organ, and relieve the congestion in those parts still in the first stage of disease. Second, to prevent a return of this determination, and cause the matter infiltrated or effused in parts farther advanced in disease, to be absorbed or expectorated. And third, to keep up an active state of those secretions, which by their highly carbonaceous composition, may in some manner compensate for the diminished function of respiration. To this class belong the hepatic, intestinal, and cutaneous secretions. If these objects can be fully secured, complete resolution of the inflammation will soon follow. As there is nothing in the case before us to forbid it, I shall attempt to accomplish the first object by bleeding from the arm to an extent sufficient to lesson the pain, diminish the febrile heat of the skin, and remove the great sense of oppression and tightness across the chest, whether the quantity required be one pint or two. I bleed such a case not to take away a given *quantity* of blood, but to produce a given effect on the circulation. To keep up the sedative effect induced by the bleeding, and to favor the absorption of infiltrated and effused matters into the pulmonary tissue and pleural cavity, I shall follow the bleeding immediately by full nauseating doses of a solution of Tartrate of Antimony and Potash, repeated every two hours, with two grains of Calomel between the doses of the Antimony.

If the bowels become freely moved, as is generally the case, in the course of six or eight hours, I shall add to each alterative dose of the Calomel one grain of Opium to aid in quieting irritability and causing the Antimony to be better borne. The third indication will thus be fulfilled by the same means as the second. If, in opposition to the sedative influence of the Antimonial, the febeile symptoms again increase in activity, a few hours after the first bleeding, I shall direct the latter to be repeated under the same rules as at first. The further progress of the case and the influence of treatment we will note when we meet in the wards to-morrow morning at 9 o'clock.

Dec. 10th, 9 o'clock A. M. In carrying out the treatment directed for this patient yesterday, he was bled nearly 30 oz. before the pain, difficult breathing, and sense of oppression were relieved to the extent desired. The relief, however, from the abstraction of that quantity was very decided, although no sense of syncope ensued. The heat of the skin was diminished, the pulse more soft, and the patient capable of taking quite a full breath with but little pain. Having gained a decided impression and for the time being arrested the determination of blood to the inflamed lung, ten grains of Tart. Ant. et Pot. were dissolved in half a tumbler of cold water, of which a tea-spoonful was given every two hours and two grains of Calomel between each dose. The first spoonful of the Antimonial solution being given immediately after the abstraction of blood, caused pretty free vomiting, accompanied by perspiration and still further relief of the breathing. The subsequent doses were borne with very little nausea, and with the aid of the small doses of Calomel, operated freely as physic at the end of six or eight hours. Immediately after the free movement of the bowels, four grains of Calomel combined with one of Opium were given and the solution of Antimony alone continued up to the present time. If you now look at the patient, his face is less flushed; if you feel of the skin you will find it not much above the natural temperature; his pulse is still about 90 per minute, but soft; his breathing you perceive is much less hurried than yesterday, with pain only on coughing or taking a long breath.

If you look in the vessel here you see that the expectorated matter is still very red or bloody and abundant, but thicker than before, and the cough less frequent. Percussion shows but little alteration since yesterday, but the vesicular murmur or crepitation has entirely subsided leaving in its place only the mucus rhoncus caused by the bloody mucus in the air passages.

The patient is still thirsty and the tongue covered with a yellowish white fur. The bowels have been evacuated once this morning. Now I shall direct a continuance of the solution of Antimony every three hours, and a powder composed of Calomel 4 grs. Opii 1 gr. every six hours, with toast water for drink, and if the pain remains pretty severe in the side on coughing, a blister must be laid over the affected part this evening.

Dec. 11th, 9 o'clock A. M. Now gentlemen, you see the breathing of this patient is quite easy, his skin cool, his pulse not over 85 per minute and soft; he expectorates quite easy, the sputa being thicker and much less bloody; on taking a forced inspiration, the affected lung fills more readily and with little pain; the coat on the tongue is loosening along the edges and is moist; there is a slight mercurial fetor to the breath; and the bowels have been opened once or twice during the night, The blister mentioned yesterday was applied last evening and has drawn well.

I shall now give the patient nothing but a solution of Antimony with the addition of five or six drops of Laudanum to each dose, repeated every three or four hours. A little gruel and mucillages may be allowed during the day.

Dec. 12th, 9 o'clock A. M. You will see in examining this patient now, that all his symptoms are still further improved. His skin is cool; tongue moist and nearly clean; the expectoration is less copious, more thick, and nearly destitute of redness; his breathing while quiet, is easy, but still the left side of the chest expands less freely than the other, and you perceive as we apply percussion, that there is considerable dulness, though occupying a smaller space than at first. These circumstances show that the inflammation proper has nearly subsided, and what remains to be done is to cause a removal, by absorption, of its consequences. For this a continuance of external irritation by repeating the blister, the exhibition of the Compound Honey of Squills, Senega, and Antimony with Paregoric to allay the cough; keeping the bowels regular by the mildest laxatives; and as the patient begins now to manifest some appetite, restricting him to a very simple diet consisting of soft boiled rice, weak animal broths &c., will be all that is needed to complete the cure.

14th, 9 o'clock A. M. Here is our patient, lying in bed reading a newspaper. A glance at his general aspect is sufficient to show that he is rapidly improving. The respiratory murmur has returned over the greater part of the affected lobe of the lung; he coughs little and the sputa are thick, yellow, and entirely destitute of blood; but still there is some dullness at the lower margin of the chest, and a forcible inspiration gives a slight sense of soreness. We shall continue the same treatment mentioned on the 12th inst.

Dec. 16th. Patient sitting up with a good appetite, and appears entirely convalescent. No further medical treatment required.

There gentlemen, is a case which one week since presented all the phenomena of an acute Pleuro-Pneumonia, in the fourth day of its progress. Now the patient is well. [He was discharged well on the 19th, three days after.] Yet you will find some high authorities in Medicine, who will tell you that Pneumonia cannot be cut short by bleeding, and will array abundance of numerical statistics, gathered up promiscuously without reference to the class of patients or their previous tone of vitality, as evidence of their assertion. But gentlemen, such evidence is scarcely worth a serious examination. Pneumonia, as in the case before us, may be characterized by an active and vigorous state of the functions of innervation and circulation, when little else than a judicious application of bleeding and antimony is required to subdue it; or it may be accompanied by an enfeebled condition of these functions, manifested by an early appearance of dullness and dinginess of the face, a more rapid and easily compressed pulse, and by a less forcible or sustained impulse of the heart, in which the same amount of bleeding and Antimony would speedily induce a dangerous, if not fatal degree of prostration, or an equally dangerous degree of intestinal irritation. But we shall have abundant opportunities to illustrate these varieties in our wards before the present term expires.

I intended to embrace in this report two or three other cases, but I have already filled more space than is desirable, and yet have not done justice to the very minute and excellent practical instruction of the attending physician.

